# Quaternary Ammonium-Based Ionosilica Hydrogels as Draw Solutes in Forward Osmosis

**DOI:** 10.3390/molecules25245987

**Published:** 2020-12-17

**Authors:** Alysson Duarte Rodrigues, Matthieu Jacob, Véronique Gauchou, Jean-Olivier Durand, Philippe Trens, Peter Hesemann

**Affiliations:** 1 Institut Charles Gerhardt, Université de Montpellier—CNRS, 34090 Montpellier, France; alysson.duarte-rodrigues@umontpellier.fr (A.D.R.); jean-olivier.durand@umontpellier.fr (J.-O.D.); philippe.trens@enscm.fr (P.T.); 2 Pole D’Etude et de Recherche de Lacq (PERL), Pôle Economique 2, BP 47-64170 Lacq, France; veronique.gauchou@total.com

**Keywords:** ionosilica, hydrogel, forward osmosis, draw solute

## Abstract

In the last few years, forward osmosis (FO) has attracted increasing interest as a sustainable technique for water desalination and wastewater treatment. However, FO remains as an immature process principally due to the lack of efficient and easily recyclable draw solutes. In this work, we report that ionosilica hydrogels based on quaternary ammonium halide ionosilica are efficient draw solutes in FO. Fluidic ionosilica hydrogels were obtained via hydrolysis-polycondensation reactions of a trisilylated quaternary ammonium precursor in slightly acidic water/ethanol solvent mixtures. The liquid-to-gel transition of the precursor and the kinetics of the formation of hydrogels were monitored by liquid NMR measurements. The formed hydrogels were shown to generate osmotic pressure up to 10.0 atm, indicating the potential of these hydrogels as efficient draw solutes in FO. Our results suggest that iodide anions are the osmotically active species in the system. Regeneration of the hydrogels via ultrafiltration (UF) was successfully achieved, allowing the development of a closed FO-UF process. However, the osmotic performances of the ionosilica hydrogels irreversibly decreased along the successive FO-UF cycles, probably due to anion exchange processes.

## 1. Introduction

Energy and water scarcity are among the most challenging current problems, with huge societal and environmental consequences. In 2010, half a billion people lived in water-stressed or water-scarce countries or regions, and by 2025 that number will grow up to three billion [1]. Growing population, increasing water demand and energy consumption currently stimulates the exploration of more sustainable strategies for water upgrading and treatment. The development of novel techniques for water purification and desalination is of particular interest and contributions for less energy consuming methods, even for niche technologies, are highly desirable.

In this context, forward osmosis (FO) has attracted considerable interest in the last few years as a sustainable water desalination method [2,3,4]. FO utilizes the osmotic pressure gradient between a feed and a draw solute as a driving force. The physical phenomenon of FO can simply be defined as the movement of water molecules across a semipermeable membrane due to difference of the osmotic pressure between a feed and a draw solution. The semipermeable membrane allows only water molecules to permeate through while it retains the solute molecules [5]. FO has a range of potential advantages. Due to the application of low or no hydraulic pressure, FO is potentially less energy consuming than conventional desalination technologies such as reverse osmosis (RO) or distillation. In addition, FO desalination results in lower and reversible membrane fouling. FO desalination should also considerably reduce the quantity of brine generated from desalination plants [6]. FO is therefore a promising and economic alternative to conventional desalination processes.

However, despite considerable recent progress in the area of membrane elaboration [7], FO remains as an immature technology due to the lack of efficient and recyclable draw solutes. An ideal draw solute (DS) should generate high water flux, low back flux, and allow for an efficient and inexpensive recovery [8,9,10]. Various draw solutes such as inorganic and organic salts, hydrophilic nanoparticles, dissolved gases or volatiles or switchable polar solvents have been investigated [2,11,12]. All systems display distinct advantages and drawbacks, but none of them combines the required profiles allowing economic large-scale applications. The development of novel and more performant DS is still a challenge and is therefore intensively studied.

Ionic liquids are among the draw solutes that have recently been taken into account as draw solutes in FO [13], but their application is limited due to their problematic regeneration procedures. Some recent investigations have therefore focused instead on polyelectrolytes [14] and poly(ionic liquids) [15] as draw solutes, allowing for simpler recovery and regeneration procedures. In this context, the use of polymer hydrogels was also considered [16,17,18]. Most of the studied hydrogels are based on organic polymers such as polyacrylates, poly(acrylamides), poly(N-isopropylacrylamide) (PNIPAM) or polystryrene sulfonates. These systems appeared as stimuli-responsive systems allowing easy recovery via thermal responsiveness [16,18]. Hydrogels therefore combine good performance in generating high osmotic pressure and easy regeneration [18].

Here, we have investigated for the first time ionosilica hydrogels as draw solutes in FO. As a silica-supported form of ionic liquids, ionosilicas recently appeared as highly polyvalent materials with high potential for applications in separation [19,20,21,22], catalysis [23,24] and drug delivery [25,26]. The high hydrophilicity of ionosilicas was monitored via solvent adsorption measurements [27]. Indeed, ionosilicas are relatively inexpensive materials that can be obtained in a kilogram scale from standard chemicals such as (3-chloropropyl)trimethoxysilane and (3-aminopropyl)trimethoxysilane. The availability of ionosilicas in large quantities allows applications of these phases on a pilot scale in chromatography, anion exchange and thermochemical heat storage [28]. Ionosilicas are therefore not only laboratory curiosities, but easily accessible and highly polyvalent materials with a broad application spectrum.

In this work, we evaluated the potential of ionosilicas as draw solutes in FO. Ionosilica hydrogels can be considered as the mineral counterpart of polymer hydrogels. In the area of sustainable chemistry, silica is a support of choice due to its non-toxicity and biocompatibility. The sol-gel process allows an easy access to tailor silica hydrogels with tunable properties. The use of ionic silylated precursor molecules allows accessing ionosilica hydrogels incorporating a high number of hydrophilic ionic sites. Due to their easy synthesis, we were able to investigate FO processes with ionosilica hydrogel draw solutes directly in a semi-industrial FO pilot. Ultrafiltration (UF) was employed to recycle the dispersions after FO and to regenerate the hydrogels in view of a closed FO-UF cycle.

## 2. Results and Discussion

### 2.1. Synthesis and Characterization of the Ionosilica Hydrogel

**Scheme 1:**
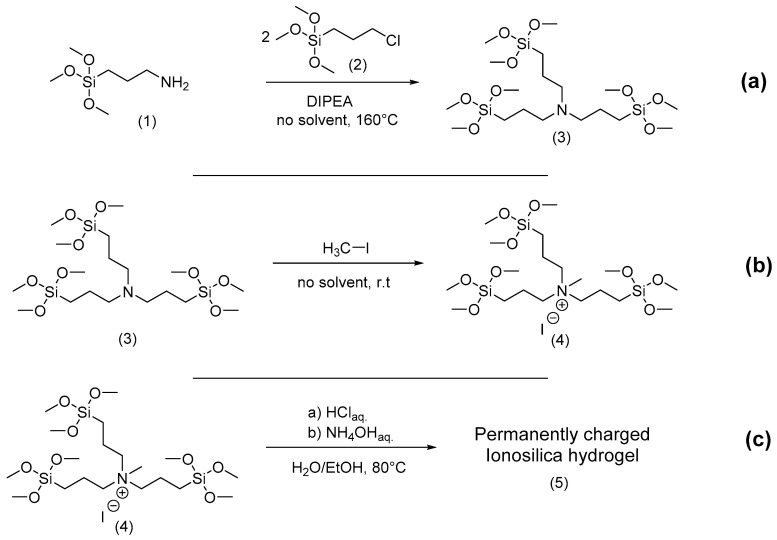
Reaction sequence for the formation of the ionosilica hydrogels. (**a**) Formation of the *tris*-trimethoxysilylated amine **3**; (**b**) methylation of **3** to yield the quaternized ammonium precursor **4**; (**c**) Sol-gel transformation of precursor **4** to yield permanently charged ionosilica hydrogels.

The reaction sequence for the formation of the ionosilica hydrogels is illustrated in Scheme 1a–c. The quaternary ammonium precursor was obtained following published procedures [25,29,30]. A straightforward three step sequence starting from 3-aminopropyl trimethoxysilane (**1**) and 3-chloropropyl trimethoxysilane (**2**) led to in a first time to the *tris*-trimethoxysilylated amine precursor **3**. The subsequent methylation of compound **3** using methyl iodide yielded the trisilylated quaternized precursor *N*-methyl-*N*,*N*,*N*-*tris*(3-(trimethoxysilyl)propyl) ammonium iodide (**4**, Scheme 1). The permanently charged ionosilica hydrogel **5** was synthesized from **4** via hydrolysis-polycondensation reactions in aqueous media under relatively mild reaction conditions.

The first reaction step, the alkylation of (3-aminopropyl)trimethoxysilane, was carried out by mixing (3-aminopropyl)trimethoxysilane (**1**), (3-chloropropyl)trimethoxysilane (**2**) and *N*,*N*-diisopropyl ethylamine (DIPEA). The reaction mixture was heated to 160 °C for 36 h. The alkylation reaction was followed by ^1^H-NMR in the liquid phase. At the end of the reaction, a color change together with the formation of two immiscible liquid phases could be observed. After filtration, solvent evaporation, washing with diethyl ether and distillation under reduced pressure we obtained the *tris*(trimethoxy)silylated amine **3** in high yield (83%). In a second reaction step, this compound was used for the quaternization reaction with methyl iodide to give the trisilylated ammonium precursor *N*-methyl-*N*,*N*,*N*-*tris*(3-(trimethoxysilyl)propyl) ammonium iodide (**4**) in quantitative yield. This compound is indeed an ionic liquid: it appears as a highly viscous liquid at room temperature. Due to its low crystallinity and negligible vapor pressure, the purification of the precursor **4** can only be carried by washing procedures with selected solvents (alkanes, diethyl ether), followed by solvent evaporation.

This precursor **4** was then used for the formation of ionosilica hydrogels via controlled hydrolysis-polycondensation procedures under slightly acidic conditions. In a typical experiment, precursor **4** was solubilized in acidified water/ethanol mixture under heating to 80 °C, thus yielding visually transparent ionosilica hydrogels. The synthesis was carried out under acidic conditions (pH = ~2) favoring faster hydrolysis prior to heating. The slight increase of pH to 5, by addition of few drops of diluted aqueous ammonia solution to the reaction mixture, favored the formation of reticulated hydrogels. Finally, the organic solvent (ethanol) was evaporated, leading to aqueous ionosilica hydrogel dispersions.

**Figure 1:**
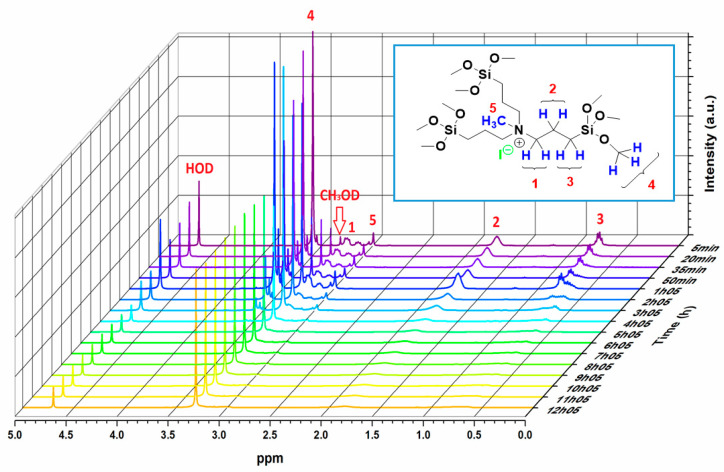
^1^H-NMR kinetic study of the hydrolysis of compound **4** in D_2_O.

Liquid ^1^H-NMR analysis in D_2_O was used to follow the hydrolysis-polycondensation reaction of the precursor **4** to form the ionosilica hydrogels **5**. For these experiments, the sol-gel transition was performed at room temperature and without any other additive. Figure 1 shows the liquid ^1^H-NMR spectra of the reaction mixture from 5 min to 12 h after the beginning of the reaction (c = 14.56 mmol/L). The spectra show the decrease of intensities of all ^1^H-NMR peaks attributed to the molecular structure of the compound **4** in solution. After 3 h, the spectra only display the signals of residual water (from D_2_O) and methanol, formed via the hydrolysis of the methoxysilyl groups. The complete disappearance of proton signals originating from compound **4** indicates that this compound is no longer in solution, and thus confirms the formation of an aqueous dispersion of the polymeric ionosilica hydrogel.

**Figure 2:**
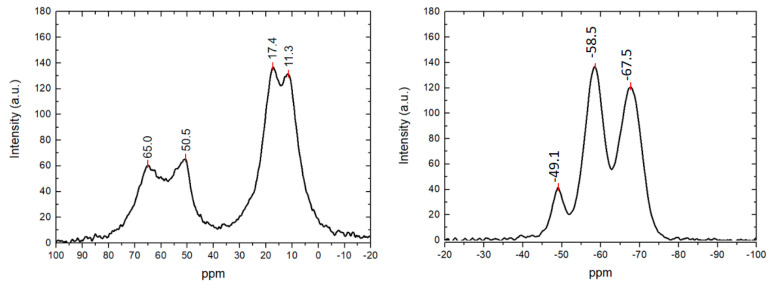
Solid-state NMR spectra of a dried hydrogel sample; left: ^13^C CP-MAS spectrum; right: ^29^Si OP-MAS spectrum.

The ionosilica hydrogels were then characterized in order to attest the constitutional integrity of the organo-ionic entities within the ionosilica hydrogel after the hydrogel formation. For this purpose, the ionosilica hydrogel **5** was air-dried and characterized by solid state NMR spectroscopy. The cross-polarization magic angle spinning (CP-MAS)-{^1^H}-^13^C and the ^29^Si OP-MAS (OP = one pulse) spectra (Figure 2) give information about the constitution of the organic structure of the hybrid material and the condensation degree of the silica network, respectively.

In the ^13^C CP-MAS analysis (Figure 2, left) we can identify the four different types of carbon centers at *δ* = 11.3, 17.4, 50.5 and 65.0 ppm, present in the molecular structure of **4**, corresponding to the bridging propyl units and the methyl group linked to the nitrogen center (Scheme 1b). These results indicate that no structural modification of the organic part of the silylated ammonium precursor occurred during the synthesis process, in agreement to our former works [25,26,29,30]. The ^29^Si OP-MAS spectrum (Figure 2, right) indicates the presence of three types of silicon centers in the material: the T^1^ band (δ = −49.5 ppm) stands for germinal silanol groups (RSi(OSi)(OH)_2_), the T^2^ band (δ = −58.5 ppm) for single silanols (RSi(OSi)_2_OH) and T^3^ band (δ = −67.5 ppm) can be attributed to fully condensed organosilica centers (RSi(OSi)_3_). Deconvolution of the spectra using the DMFit software [31] allows getting information of the condensation degree of the silica network. In the case of the hydrogel **5**, the calculated condensation degree was of 45% [32]. The hydrogel is characterized by a relatively wide-mashed, flexible and moderately reticulated ionosilica network. Finally, as no Q band was observed in the during the ^29^Si OP-MAS spectra, we can assume that no C-Si bond cleavage occurred during the synthesis process. These results are also in agreement with our previous works. [25,29,30]

**Figure 3:**
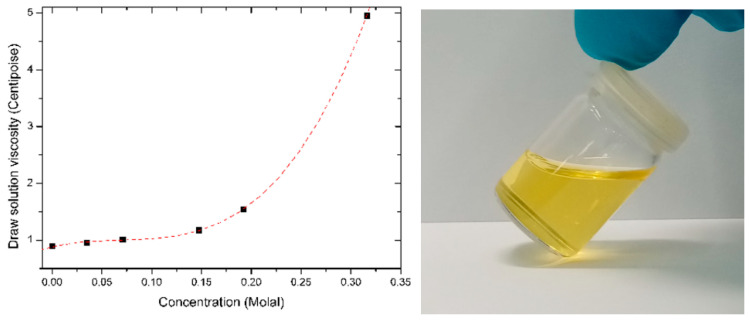
(**Left**): Viscosity of the ionosilica hydrogels at various concentration; (**right**): Photograph of a fluidic dispersion of ionosilica hydrogels.

Viscosity measurements (Figure 3) of ionosilica hydrogel dispersions having diluted iodide anions were performed using the falling ball method. Hydrogel dispersions with concentrations in the range of 0 M (pure DI water) and 0.32 M of precursor **4** were prepared via overnight stirring of the precursor **4** in DI water. Whereas at low concentration no effect was observed, a significant increase in solution viscosity was noted above approximately 0.17 M, due to the polycondensation process of precursor **4** at higher concentrations. It has to be noted that an osmolarity of approximately 1000 mOsmol/kg must be exceeded for an application of this technology for seawater desalination [33]. The solution viscosity above this amount remains relatively low (around 1.3 cP), suggesting that ionosilica hydrogel dispersions may be suitable for desalination processes.

**Figure 4:**
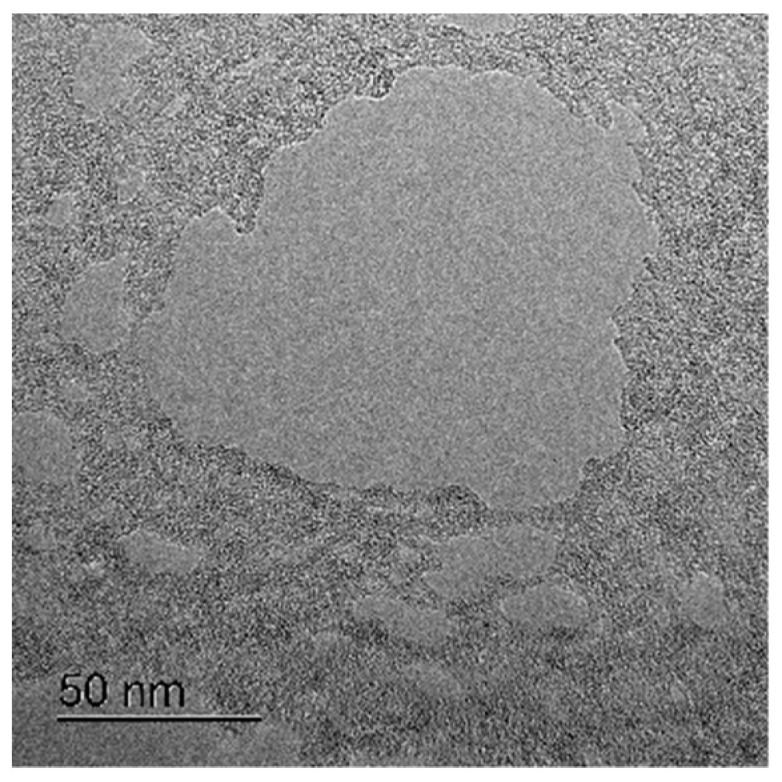
TEM image of an air-dried ionosilica hydrogel dispersion.

Finally, the TEM image of an air-dried ionosilica hydrogel (Figure 4) shows a material with a highly porous texture, indicating the formation of a homogeneously dispersed and highly hydrated material in aqueous media.

### 2.2. Forward Osmosis Experiments

**Figure 5:**
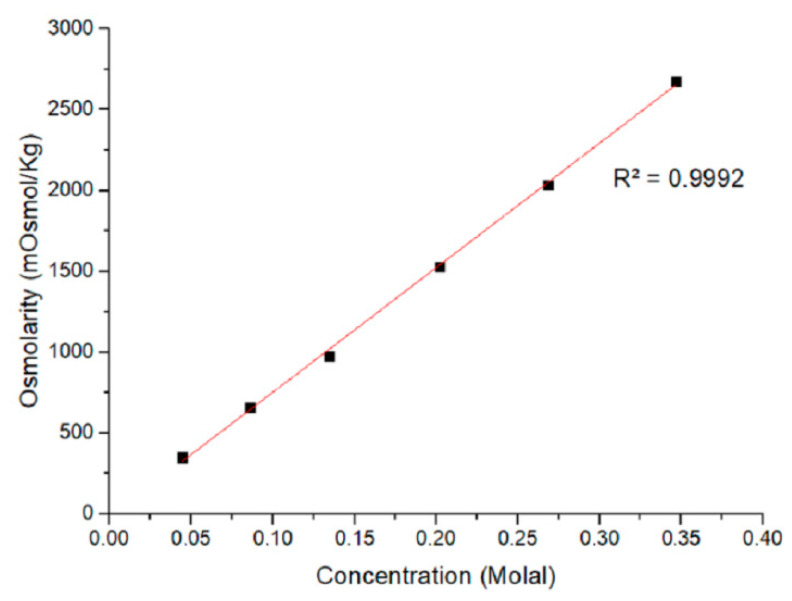
Osmolarity measurements with the ionosilica hydrogel dispersions at various concentrations.

The osmotic activity of the ionosilica hydrogel draw solute was then evaluated by osmometry with a series of hydrogel dispersions of known concentrations (Figure 5). The osmotic potential of the hydrogel dispersions was studied in a range of 0 to 0.36 molal (m) in seven distinct experiments. A linear fit of the data gave a good correlation (r^2^ = 0.9992) between concentration and osmolarity. Osmolarities above 0.36 m were not obtained because the solutions failed to freeze during the course of the analysis. Osmolarity correlation to solution concentration and FO water fluxes suggested that these data may be used to characterize draw solutions over the course of FO experiments.

**Figure 6:**
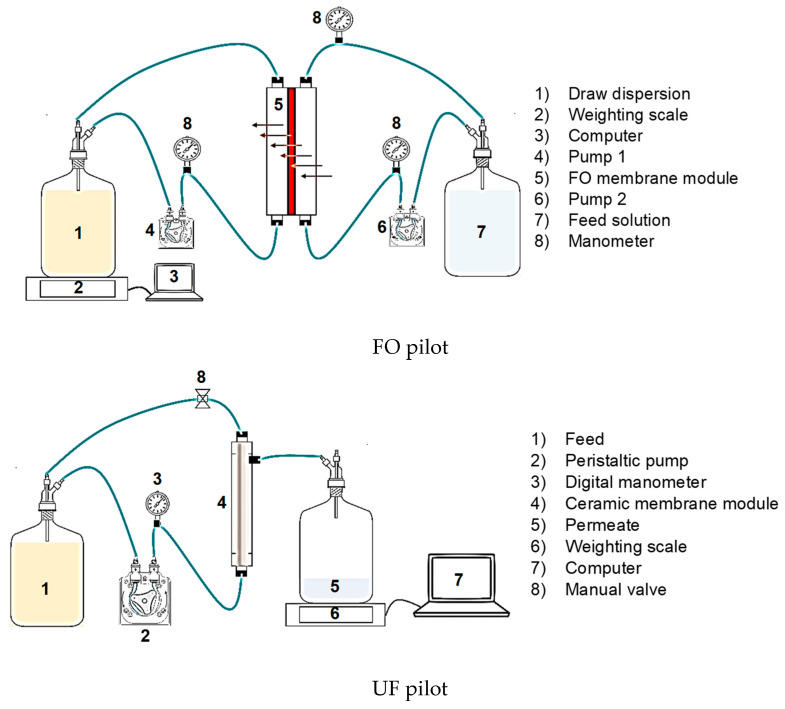
Schematic illustrations of forward osmosis (FO-) (**top**) and ultrafiltration (UF-) pilots (**bottom**).

In a second time, pilot tests were run to evaluate the osmotic performance of ionosilica hydrogel dispersions as draw solutes using a 0.1 g·L^−1^ NaCl_aq._ feed. The semi-industrial FO pilot used in this study is schematized in Figure 6, top.

Feed and draw solutions were allowed to contact the FO cellulose triacetate membrane, which was installed with the active side towards the feed solution. Production of water through FO was measured gravimetrically and recorded by weighting scale connected to a computer. Prior to the FO experiment, the feed and draw solutions were circulated in the system at room temperature (around 18 °C) until stabilization of circulating pressures.

Ionosilica hydrogel dispersions of different concentrations (containing 19.4, 12.9 and 9.7 g·L^−1^ of dried mass of DS, respectively) were tested over a two-hour period. FO water fluxes (in L·h^−1^·m^−2^) were calculated directly from plots of recovered mass of water (in grams) as function of time (min).

**Figure 7:**
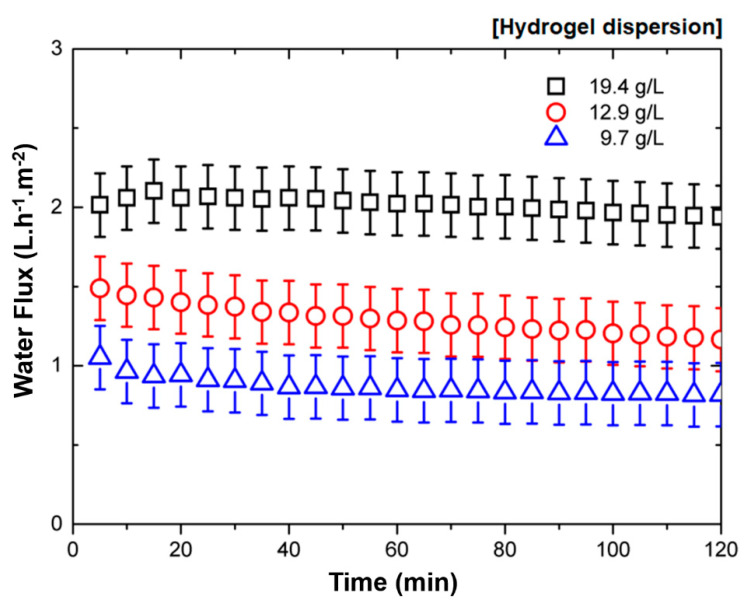
Generated water flux at various hydrogel concentrations.

Figure 7 shows averaged FO water fluxes as function of time for ionosilica hydrogel dispersions of different concentrations in water. DS with hydrogel concentrations of 19.4, 12.9 and 9.7 g·L^−1^ showed average FO water fluxes of 1.99, 1.29 and 0.87 L·h^−1^·m^−2^, respectively, in two hours of tests. Water fluxes slightly decreased over time, which may correlate to a loss of osmotic pressure with dilution of the DS. Results seem to be stable over the two hours of tests having a linear correlation (r^2^ = 0.9996) with the hydrogel concentration in the dispersion. This result clearly shows that ionosilica hydrogel dispersions generate osmotic pressure and draw water through FO. The generated water flux depends on the amount of hydrogel in the draw solution: the higher the hydrogel concentration, the higher the generated water flux. The relatively constant water flux over two hours of experiment indicates that ionosilica hydrogels did not promote fouling of FO membrane.

These results indicate that the water fluxes generated by ionosilica hydrogels are twice as high as those generated by hydrogels based on organic polymers such as poly(*N*-isopropylacrylamide) (PNIPAM) or poly(sodiumacrylate) (PSA). Furthermore, these latter systems show a more pronounced decrease over time of the generated water flux compared to ionosilica hydrogels [34].

**Figure 8:**
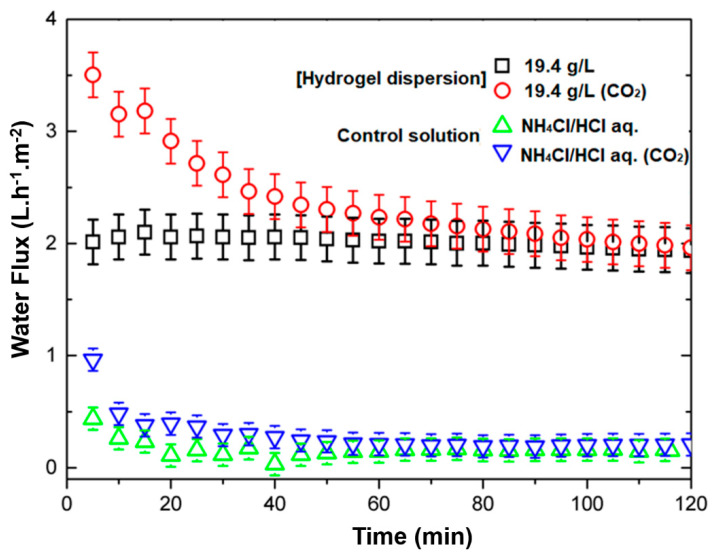
Generated water fluxes in two hours of FO tests of hydrogel dispersions and controls with and without continuous CO_2_ bubbling in draw tanks. Black squares: 19.4 g·L^−1^ hydrogel dispersion (initial pH = 3.07 and conductivity = 2.85 mS·cm^−1^) without CO_2_ bubbling. Red circles: Freshly prepared 19.4 g·L^−1^ hydrogel dispersion with CO_2_ bubbling—0.1 L·h^−1^. Green triangles: NH_4_^+^Cl^−^/HCl control solution (initial pH = 3.09 and conductivity = 2.92 mS·cm^−1^) without CO_2_ bubbling. Blue inverse triangles: Freshly prepared NH_4_^+^Cl^−^/HCl control solution without CO_2_ bubbling.

It has to be mentioned that due to the synthesis strategy, the presence of residual quantities of ammonium chloride and hydrochloric acid cannot be excluded. These two components may act as draw solutes and therefore may contribute to the generated osmotic pressure of the hydrogels. In order to evaluate the influence of these impurities on the osmotic properties, control experiments using similar salt/acid concentrations (with similar pH and conductivity values) were run in the absence of ionosilica hydrogel dispersions and compared with experiments in the presence of the ionosilica hydrogels. We also studied the influence of carbon dioxide bubbling on the osmotic properties of hydrogel dispersions. The results of these two parameters are summarized in Figure 8.

As already discussed, the ionosilica hydrogel dispersions generate significant water flux. As an example, the sample containing 19.4 g/L ionosilica hydrogel dispersion in water (pH = 3.07; conductivity = 2.85 mS·cm^−1^) generated an average water flux of 1.99 L·h·m^2^ (Figure 8—black squares). The generated water flux is nearly constant during the whole experiment time (120 min). On the other side, the control test (Figure 8—green triangles) with an aqueous NH_4_^+^Cl^−^/HCl solution ([NH_4_Cl]_initial_ = 2.8 mM/[HCl]_initial_ = 7.5 mM) ran in similar pH and conductivity values (pH = 3.09; conductivity = 2.92 mS·cm^–1^) resulted in significantly lower average water flux of 0.2 L·h·m^2^. These results indicate that the presence of ammonium chloride and hydrochloric acid only marginally influences the osmotic performances of the dispersions. The performances are however still far from the competing RO technology that can produce around 15–30 L·h·m^2^.

To improve osmotic performances of hydrogel dispersions and knowing that ammonium cations from ionosilica structure could allow anionic interactions, we tested the influence of carbon dioxide (CO_2_) bubbling in draw tanks during FO process. CO_2_ (g) was used to increase the number of osmotically active species of the system by the formation of carbonates or bicarbonates. Furthermore, CO_2_ bubbling allows controlling the pH of the dispersion along the FO process.

In a typical test, a freshly prepared ionosilica hydrogel dispersion containing 19.4 g·L^−1^ (Figure 8—red circles) was exposed to a 0.1 L·h^−1^ bubbling of CO_2_ (g) during the whole experiment (2 h). The resulting dispersion showed improved water flux in the first 95 min of the test, reaching in best water flux results 3.5 L·h^−1^·m^2^. This increased water flux probably originates from the increase of osmotically active species in the draw solution. However, the water flux decreased rapidly and reached the values observed with ionosilica hydrogels without CO_2_ treatment after approximatively 120 min (Figure 8—black squares). This decline can be explained by the loss of CO_2_ from the draw solution. Indeed, the dissolved CO_2_ do not seem to interact sufficiently with the hydrogel and quickly degasses from the open system. Overall, CO_2_(g) bubbling is a good alternative to maintain the pH of the dispersion stable (pH = ~5) in the course of the FO process, but as the CO_2_ degasses quickly from the draw solution, the generated osmotic properties decrease significantly and reach the initial value after approx. 120 min.

In a control test, we also studied the osmotic properties of dissolved CO_2_ in the absence of ionosilica hydrogel (Figure 8—blue inverse triangles). Our results show that CO_2_ degasses more quickly from the aqueous draw solution in the absence of hydrogels, as the complete elimination of CO_2_ is finished after approx. 40 min. The ionosilica matrix seem to retain the dissolved CO_2_ in solution and prevent its elimination.

### 2.3. UF Regeneration of Ionosilica Hydrogel Dispersions

The aim of our study was the development of a closed FO-regeneration cycle. We therefore studied the regeneration of the ionosilica hydrogel after the FO process. Ultrafiltration (UF) was used to regenerate the hydrogel dispersions in a separated UF set-up once FO process was finished.

**Figure 9:**
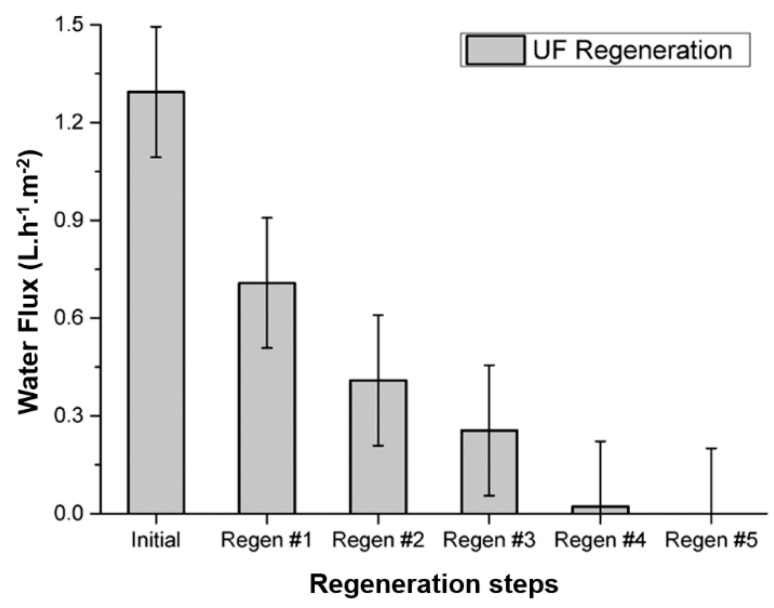
Generated water flux in successive FO/UF cycles with a 12.9 g·L^−1^ ionosilica hydrogel dispersion.

The UF setup is schematically illustrated in Figure 6, bottom. In a typical UF procedure, the initial ionosilica hydrogel (typically containing 12.9 g·L^−1^ of the hydrogel) was introduced in the UF module and different volumes of water were tangentially filtered using a ceramic UF membrane. Prior a new FO cycle, DI water was added restituting initial volumes of the dispersions. These ionosilica hydrogel dispersions were then used for a new FO process. In this way, the ionosilica hydrogels were used in up to five successive FO-UF cycles. The results are depicted in Figure 9.

Results from Figure 9 show that FO water fluxes of a 12.9 g·L^−1^ ionosilica hydrogel dispersion generated osmotic pressure, and resulted in a water flux of 1.29 L·h^−1^·m^2^ in the first cycle. However, starting from the second FO cycle, the water flux decreased considerably to 0.71 L·h^−1^·m^2^. This value further decreased in the following FO-UF cycles and tended to zero after the fifth regeneration. This behavior could be attributed to a chemical change of the hydrogel, i.e., the loss of the osmotically active anions via anion exchange processes, thus resulting in decreased osmotic performances of the hydrogel dispersions. Indeed, our results indicate that ionosilica hydrogels generate osmotic pressure due to the presence of anions (I^−^ or Cl^−^). Iodide anions seem to be mobile in the hydrogel dispersions and were designed as the main osmotic vector of the system. In the course of FO-UF cycles, iodide was probably irreversibly exchanged from ionosilica structure against osmotically less active species such as bicarbonates. However, treatment of ionosilica dispersions with diluted hydrochloric acid (pH = 2.25) after the fifth FO-UF cycle did not increase the osmotic properties of the materials. The absence of osmotic potential of HCl re-acidified dispersions (with FO water flux = 0 L·h^−1^·m^2^) supports the idea that iodide anions are the main osmotic vector of the system, and we anticipated that the loss of iodide anions irreversibly results in decreasing osmotic properties of the hydrogels. We therefore determined the iodide content of the hydrogel dispersions and the UF-filtrate after each regeneration cycle (Table 1).

The loss of osmotically active iodide anions from draw solutes into the produced water was evaluated by ion chromatography (IC). For this purpose, the iodide concentrations in the initial draw dispersion containing 12.9 g·L^−1^ of the ionosilica hydrogel and after five FO/UF cycles were measured. The iodide content in the draw solution decreases from 14.01 mM to 9.27 mM after the fifth FO/UF cycle, which represents the loss of approximately one third of the iodide anions in five FO/UF cycles. On the other hand, we also determined the iodide content in the feed solution after each FO/UF cycle. The determination of the iodide content of the filtrate confirms that iodide leaches out, as 2.53 mM and 1.88 mM of iodide were detected in the filtrates after the first two FO/UF cycles, respectively. However, the amount of released iodide strongly decreases in the course of the following cycles. The total amount of released iodide is of 4.5 mM in the five FO/UF cycles, that is in agreement with the decrease of the iodide content in the draw dispersion. However, these results indicate that a significant amount of iodide is still present in the draw dispersion after five FO/UF cycles. The decrease of the osmotic properties therefore is not exclusively related to the loss of osmotically active iodide anions or by its exchange with other anionic species, but other parameters have to be taken into account. We suggest that these results may be explained by a textural evolution of the hydrogels, i.e., agglomeration procedures that limit the mobility of the osmotic vectors within the hydrogel matrix.

## 3. Materials and Methods

### 3.1. General

The permanently charged precursor *N*-methyl-3-(trimethoxysilyl)-*N*,*N*-bis(3-(trimethoxysilyl)-propyl)propan-1-amonium iodide was synthesized following an adapted procedure with commercial reagents without solvents in standard and oven dried Schlenk flasks under argon. Concentrated hydrochloric acid (37%, Sigma-Aldrich, Saint Louis, MO, USA) and ammonia solution (30%, Sigma-Aldrich) were diluted in deionized (DI) water before use. Reticulated hydrogels were synthesized in DI water and absolute ethanol (>99.9% purity, VWR, Radnor, PA, USA) solvent mixtures and commercial reagents have been used without any further purification.

### 3.2. Characterizations

Liquid NMR spectroscopic data was obtained with an Advance 200 (routine) or 400 MHz spectrometer (Bruker, Billerica, MA, USA) and chemical shifts are quoted in parts per million (ppm) relative to tetramethylsilane (TMS). Coupling constant (*J*) values are given in Hertz. Solid NMR data were obtained with a Cryomagnet (Oxford Instruments, Oxford, United Kingdom) spectrometer working at 300 MHz with around one gram of dried samples using cross polarization magical angle spinning—CP-MAS-(^13^C and ^29^Si) and MAS ^29^Si (one-pulse) techniques. High performance liquid chromatography (HPLC) was performed on an Alliance 2795 system (Waters Cooperation, Milford, MA, USA) equipped with a Waters ESI-QTOF mass spectrometry (MS) detector using electro spray ionization (ESI) methods in high and low resolutions. Elemental analysis was performed using a FlashEA^®^ 1112 system (Thermo Scietific, Waltham, MA, USA). FT-IR measurements were recorded on a Spectrum Two FT-IR spectrometer (Perkin Elmer, Waltham, MA, USA). SEM images were obtained on a S4800 FS instrument (Hitachi, Tokyo, Japan) and TEM images were obtained on a 2200 FS—200 kV system (JEOL, Tokyo, Japan) equipped with a CCD USC 4092 × 4092 px^2^ camera (Gatan, Pleasanton, CA, United States). Osmometric measurements were realized with a freezing point osmometer (Osmomat 3000, Gonotec, Berlin, Germany) with 50 μL samples. Samples were homogenized by aid of an orbital shaker (300 rpm) during one night before analysis. Viscosity measurements were performed using a Lovis 2000 ME micro-viscosimeter (falling ball method, Anton Paar, Graz, Austria), the data presented is the average of 10 individual measurements obtained at an average temperature of 25 °C.

### 3.3. Synthesis of tris(3-(trimethoxysilyl)propyl)amine (**3**)

The synthesis of the neutral amine precursor **3** was carried out following standard literature protocols [20,22,29,30]. (3-Aminopropyl)trimethoxysilane (84.5 mL, 0.36 mol, 1 equiv.), (3-chloropropyl)trimethoxysilane (232 mL, 1.28 mol, 3.5 equiv.) and *N*-ethyldiisopropylamine (248 mL, 1.46 mol, 4 equiv.) were mixed under an argon atmosphere. The light-yellow homogenous solution was stirred magnetically (600 rpm, room temperature) for 5 min prior to heating the solution at 160 °C for 30 h. After this time, the obtained biphasic light yellow/orange solution was cooled to room temperature (overnight) and then to 0 °C in an ice bath (3 h). The *N*-ethyldiisopropyl ammonium chloride precipitated and was eliminated by successive recrystallizations and filtrations. The resulting solution was purified by medium vacuum distillation (<1 × 10^−3^ mbar) starting from room temperature until 160 °C (with increments of 20 °C) until no more unreacted starting materials evaporated. The final orange liquid was distilled, yielding the title compound as a colourless liquid. Yield = 83%. (Density: 1.0904 g/cm^3^). ^1^H-NMR: (CDCl_3_, 400 MHz) *δ* (ppm): 3.34 (s, 27H); 2.18 (m, 6H); 1.30 (m, 6H); 0.37 (m, 6H); ^13^C-NMR: (CDCl_3_, 150 MHz) *δ* (ppm): 57.1, 50.5, 20.2, 6.7; HRMS [ESI+] calcd. for C_18_H_46_N_1_O_9_Si_3_: 504.2480 [cation M+1]; found: 504.2480; IR-ATR (cm^−1^): 2941, 2836, 1463, 1189, 1079, 811, 779, 442. Elemental analysis: Calculated: C: 42.91; H: 9.0, N: 2.78; Found: C: 41.54; H: 8.15, N: 2.57.

### 3.4. Synthesis of N-methyl-3-(trimethoxysilyl)-N,N-bis(3-(trimethoxysilyl)propyl)propan-1-ammonium Iodide (**4**)

The synthesis of the precursor **4** was carried out by mixing the trisilylated amine precursor **3** (109.04 g, 216.6 mmol, 1 equiv.) with iodomethane (46.12 g, 324.9 mmol, 1.5 equiv.). The reaction mixture was stirred at room temperature during 16 h under argon. After completion, the crude product was washed with anhydrous diethyl ether and pentane (3 × 200 mL, 10 min each). The dark-brown and viscous compound was dried during 3 h under vacuum (10^−3^ mbar), yielding precursor **4** in quantitative yield. The isolated compound was characterized by the different techniques. ^1^H-NMR: (CDCl_3_, 400 MHz) *δ* (ppm): 3.52 (s, 27H); 3.42 (m, 6H); 3.18 (s, 3H); 1.73 (m, 6H); 0.64 (m, 6H); ^13^C-NMR: (CDCl_3_, 150 MHz) *δ* (ppm): 65.8, 50.8, 50.7, 16.1, 5.5. FT-IR-ATR (cm^−1^): 2941, 2840, 1466, 1414, 1188, 1065, 812, 781, 449. HRMS [ESI+]: HRMS [ESI+] calcd. for C_19_H_48_NO_9_Si_3_^+^ 518.2643 [cation M+1]; found: 518.2643.

### 3.5. Synthesis of Hydrogel Dispersions with Precursor **4**

A solution of precursor **4** (25 g, 39 mmol) in absolute ethanol (1 L) was added dropwise to an aqueous HCl solution (1 L H_2_O, with 2 mL of HCl 18.5%) at room temperature (600 rpm). After total addition of the precursor, the resulting homogenous yellow solution was stirred in the same conditions for 20 min. Then, 4 mL of a 12% ammonia aqueous solution were added and the final solution was heated at 80 °C for 30 h (reflux). After this time, the volatiles were evaporated and the resulting ionosilica hydrogel dispersion in water was used as draw solute without any further purification.

## 4. Conclusions

Ionosilica hydrogels could easily be obtained from quaternized trisilylated ammonium precursor. As the synthesis of the hydrogels can easily be up-scaled, we were able to obtain hydrogels in sufficient quantity for FO experiments on a pilot scale. Applications as draw solutes in FO indicated that the ionosilica hydrogels generate osmotic pressure of 10.01 atm. After the FO process, the ionosilica hydrogel draw solute can efficiently be regenerated via ultrafiltration (UF). However, regenerated hydrogels display decreasing generated osmotic pressure. This trend can be explained either by anion exchange or carbon dioxide adsorption of the draw solution, yielding osmotically inactive carbonates or bicarbonates, or by a morphological or textural evolution of the hydrogels during the FO/UF process. The hydrogels therefore show limited applicability in closed FO-UF cycles or long-lasting processes. However, this study shows that hydrogels based on mineral support also can be used as draw solutes in FO, similarly to polyelectrolytes based on organic polymeric hydrogels based on organic polymers. The finding of this study is therefore of fundamental interest for a better comprehension of ionosilica materials and their behavior at the solid/liquid interface.

## Figures and Tables

**Table 1:** Iodide concentration in the ionosilica hydrogel dispersion and UF filtrates along the FO/UF cycles. Initial ionosilica hydrogel dispersion = 12.9 g·L^−1^ (dried mass).

Sample		[I^−^] mM (± 0.2 mM)
Hydrogel dispersion	Initial	14.01
	Final—(after five FO/UF cycles)	9.27
UF filtrate	Cycle 1	2.53
	Cycle 2	1.88
	Cycle 3	0.05
	Cycle 4	not detected
	Cycle 5	not detected
